# Glucose Intolerance and the Amount of Visceral Adipose Tissue Contribute to an Increase in Circulating Triglyceride Concentrations in Caucasian Obese Females

**DOI:** 10.1371/journal.pone.0045145

**Published:** 2012-09-28

**Authors:** Margot Berings, Charline Wehlou, An Verrijken, Ellen Deschepper, Ilse Mertens, Jean-Marc Kaufman, Luc F. Van Gaal, D. Margriet Ouwens, Johannes B. Ruige

**Affiliations:** 1 Department of Endocrinology, Ghent University Hospital, Ghent, Belgium; 2 Department of Endocrinology, Diabetology and Metabolism, Antwerp University Hospital, Antwerp, Belgium; 3 Biostatistics Unit, Ghent University, Ghent, Belgium; 4 German Diabetes Center, Institute for Clinical Biochemistry and Pathobiochemistry, Düsseldorf, Germany; Postgraduate Medical Institute & Hull York Medical School, University of Hull, United Kingdom

## Abstract

**Context:**

Lipotoxicity is a risk factor for developing obesity-related metabolic complications, including non-alcoholic fatty liver disease, type 2 diabetes (DM2), cardiovascular disease and stroke. Yet, the mechanisms underlying the development of lipotoxicity itself remain poorly understood. Here, we investigated whether glucose intolerance aggravates lipotoxicity by evaluating the association between triglyceride (TG) concentrations and glucose tolerance status in a cross-sectional study on obese Caucasian women at risk for DM2.

**Methods:**

913 obese females unknown to have diabetes were recruited (mean age: 41.2±SD 12.3; median BMI: 36.2, IQR 32.9–40.2). Visceral (VAT) and subcutaneous abdominal adipose tissue volumes were quantified with computed tomography. Glucose, insulin, and triglyceride concentrations were determined in fasting state and following a 75 gram oral glucose tolerance test.

**Results:**

Based on fasting and 2 h post-load glucose levels, 27% of the women had impaired glucose tolerance (IGT), and 8% had newly diagnosed DM2. Fasting TG concentrations were similar between the IGT- and DM2-groups, and increased as compared to women with normal glucose tolerance (NGT). Even when adjusting for age, hip circumference and VAT, fasting TG concentrations remained elevated as compared to NGT. Mixed modelling analysis of post-load responses showed that TG concentrations declined more slowly in the DM2-group as compared to IGT and NGT. However, when adjusting for VAT the difference in decline between the glucose tolerance groups disappeared.

**Conclusions:**

Glucose intolerance associates with elevated fasting TG concentrations in obese Caucasian women. We propose that glucose intolerance and increased VAT reduce lipid disposal mechanisms and may accelerate lipotoxicity.

## Introduction

Lipotoxicity, or ectopic fat deposition of non-adipose tissue, may contribute to the development of metabolic complications of obesity, including type 2 diabetes (DM2), non-alcoholic fatty liver disease, cardiovascular dysfunction, and stroke [1]. Ectopic lipid deposition occurs when the lipid storage capacity of adipose tissue is insufficient [2]. Because the liver, pancreas, skeletal- and cardiac muscle, in which ectopic lipid deposition occurs, have a limited capacity for storing lipids, cellular dysfunction and death may occur in case of lipid overload [3], [4].

Insight into mechanisms underlying the development of lipotoxicity are rapidly evolving, but still incompletely understood [5], [6]. Although it seems reasonable to expect a systemic serum component, multiple studies indicate that circulating levels of non-esterified fatty acids (NEFA) are not directly related to the severity of obesity and its complications [5]. Rather, serum triglyceride (TG) concentrations might be critical for obesity-related metabolic risk assessment [7]. For example, fasting TG concentrations are importantly associated with body shape. Subjects with a large waist circumference, which is associated with increased risk for cardiovascular disease [8], have elevated TG concentrations, whereas subjects with a *large* hip circumference, which is associated with reduced risk [9], [10], have *reduced* TG concentrations [7]. Accordingly, prospective studies in healthy young men could ascribe a substantial proportion of risk for both DM2 and cardiovascular disease to an increase in TG concentrations during a five year follow-up [11], [12].

Since glucose intolerance is importantly associated with an elevated risk for DM2 and cardiovascular disease [13], we investigated whether the presence of glucose intolerance may accelerate lipotoxicity in a cross-sectional study of a population at increased risk for developing DM2, i.e. obese women. We evaluated the association between glucose intolerance and fasting TG concentrations, as well as with the course of alterations in TG concentrations after an OGTT.

## Methods

### Setting and Participants

913 obese Caucasian females not known to have DM2 were recruited from the outpatient clinic of the Department of Diabetology, Metabolism, and Clinical Nutrition of the University Hospital, Antwerp, Belgium. Patients consulted for problems with their weight. Male patients were excluded as gender and/or sex steroids affect TG [14]. Participants who were 18 years or older, were included. Patients with very high fasting triglycerides (≥7.7 mmol/L, Third Report of the National Cholesterol Education Program [15], suspected thyroid disease (thyroid-stimulating hormone <0,1 µU/ml and free T4>18,8 pmol/L; or thyroid-stimulating hormone >4 µU/ml and free T4<9,8 pmol/L), manifestly elevated liver tests (more than 5 fold the normal upper limits: aspartate transaminase >200 U/L, alanine transaminase >280 U/L, alkaline phosphatase >485 U/L, gamma-glutamyl transferase >145 U/L) and elevated high-sensitivity C-reactive protein levels (≥3.0 mg/dL may suggest the presence of a major infection) were excluded. In addition, patients using glucose or lipid-lowering medications and patients who had undergone bariatric surgery were excluded. The study was approved by the ethical committee of the Antwerp University Hospital, and the study was performed according to the standards on human experimentation in accordance with the Helsinki Declaration of 1975 as revised in 1983 with written informed consent of the participants.

### Anthropometry and Imaging

All examinations were done in the morning between 8–10 h a.m. after an overnight fast. Length, body weight and hip circumference at the levels of the trochanter major were determined in a standard medical examination. Computed tomography at the L4–L5 level was carried out to measure the cross-sectional areas of visceral abdominal adipose tissue (VAT) and subcutaneous abdominal adipose tissue (SAT) according to the technique described by Van der Kooy and Seidell et al. [16].

### Laboratory Analyses

Venous blood samples were collected in the fasting state and during the course of a glucose tolerance test at 30, 60, 90, 120, 150 and 180 minutes after the ingestion of 75 gram glucose. Plasma glucose was measured with the glucose oxidase method (on Vitros 750 XRC; Ortho Clinical Diagnostics Inc, Rochester, NY). Triglyceride levels were measured on Vitros 750XRC (Ortho Clinical Diagnostics, Johnson & Johnson, Raritan, NJ). Insulin was measured by a radioimmunoassay with the use of Pharmacia Insulin RIA (Pharmacia Diagnostics, Uppsala, Sweden). Insulin resistance was estimated using homeostasis model assessment (HOMA-IR) as described by Matthews et al [17]. Glucose status was determined according to the criteria of the American Diabetes Association [18].

### Statistical Analysis

Statistical calculations were carried out using IBM SPSS statistics version 19 (SPSS Inc an IBM company, Chicago, IL). Normal distribution of the data was verified with the Shapiro-Wilk test and graphical methods (box plots and Q-Q plots). Variables are presented as ± standard deviation or in case of non-Gaussian distribution as median (interquartile range). To show associations with fasting TG Pearson’s correlation coefficients were presented. If necessary in case of non-Gaussian distributed data, variables were normalized by transformation into their natural logarithm (Ln transformation). General linear modelling, which allows adjustment for confounding, was deployed to evaluate potential important determinants of fasting TG, including glucose tolerance status. Finally, mixed modelling, with random intercept for subjects to account for the dependency of the repeated measures, was used to analyze the glucose, insulin and TG response following glucose ingestion and to estimate fixed effects on the TG response of different variables of interest, such as VAT, and age. A P-value <0.05 was regarded as statistically significant.

## Results

Ranking the participants based on their glucose tolerance status resulted in 593 normal glucose tolerant (NGT) -subjects, 244 impaired glucose tolerant (IGT) -subjects, and 72 subjects with newly diagnosed (DM2) (Table 1). Anthropometric and biochemical data of the 3 groups are presented in Table 1. Age of the NGT group was lower as compared to IGT and DM2. Body mass index (BMI) was lower in NGT versus IGT, but similar between IGT and DM2. VAT was lowest in NGT followed by IGT and DM2. SAT and hip circumference did not differ among the three groups. Fasting insulin levels and HOMA-IR were lowest in NGT followed by IGT and DM2. Fasting TG, as well as 2 h post-load insulin- and TG concentrations were similar between IGT and DM2 and elevated as compared to NGT.

**Table 1:** Anthropometric and laboratory measurements of females subjects according to glucose tolerance status (n = 913).

	NGT (n = 593)	IGT (n = 244)	DM2 (n = 72)	P	P^NGT-IGT^	P^NGT-DM2^	P^IGT-DM2^
Age (y)	38.74 (±11.88)	44.94 (±12.06)	48.14 (±9.82)	0.001	<0.001	<0.001	0.106
Weight (kg)	98.3 (87.6–109.3)	100.9 (90.2–113.6)	104.0 (90.1–114.4)	0.006	0.017	0.077	0.883
Height (m)	1.66 (±0.07)	1.65 (±0.07)	1.62 (±0.06)	<0.001	0.087	<0.001	0.027
BMI (kg/m^2^)	35.6 (32.2–39.4)	36.9 (33.5–42.1)	39.0 (35.1–43.1)	<0.001	<0.001	<0.001	0.155
Hip circumference (cm)	120.0 (±9.9)	119.4 (±10.8)	119.0 (±12.4)	0.595	–	–	–
VAT (cm^2^)	117.0 (90.3–162.0)	156.7 (121.0–213.0)	202.5 (149.5–283.8)	<0.001	<0.001	<0.001	0.001
SAT (cm^2^)	572.1 (±133.8)	574.7 (±123.5)	587.8 (±120.0)	0.646	–	–	–
Fasting plasma glucose (mmol/L)	4.5 (4.2–4.7)	4.7 (4.4–5.2)	5.8 (5.1–6.7)	<0.001	<0.001	<0.001	<0.001
2 hour palsma glucose (mmol/L)	6.0 (5.2–6.7)	8.6 (8.1–9.4)	12.5 (11.5–14.3)	<0.001	<0.001	<0.001	<0.001
Fasting plasma insulin (pmol/L)	93 (65–136)	121 (72–158)	158 (107–208)	<0.001	<0.001	<0.001	0.004
2 hour plasma insulin (pmol/L)	438 (265–782)	861 (488–1356)	753 (502–1435)	<0.001	<0.001	<0.001	0.998
HOMA-IR	2.5 (1.7–3.8)	3.5 (2.2–5.1)	5.8 (3.8–8.7)	<0.001	<0.001	<0.001	<0.001
Fasting plasma TG (mmol/L)	1.3 (0.9–1.8)	1.6 (1.2–2.1)	1.6 (1.3–2.4)	<0.001	<0.001	<0.001	0.973
2 hour plasma TG (mmol/L)	1.1 (0.8–1.5)	1.3 (1.0–1.8)	1.3 (1.1–2.1)	<0.001	<0.001	<0.001	0.590

VAT, visceral abdominal adipose tissue; SAT, subcutaneous abdominal adipose tissue, HOMA-IR, homeostasis model assessment (calculated insulin resistance); NGT, normal glucose tolerance; IGT, Impaired Glucose Tolerance; DM2, newly diagnosed type 2 diabetes.

Data are presented as mean (±SD) or median (25^th^ percentile–75^th^ percentile), in case of non-Gaussian distribution. Statistics according to ANOVA with Tukey correction for multiple comparisons. P, P value for overall difference between women with NGT, IGT and DM2; P^NGT-IGT^, P-value for difference between women with NGT and women with IGT; P^NGT-DM2^, P-value for difference between women with NGT and women with DM2; P^IGT-DM2^, P-value for differences between women with IGT and women with DM2.

To identify additional determinants, apart from the glucose tolerance status, of fasting TG concentrations, correlation coefficients were calculated. The strongest correlate appeared to be VAT, and then HOMA-IR (Table 2). Subsequently, multivariate linear regression analysis confirmed the independent association between fasting TG levels (dependent variable) and the glucose tolerance status, after adjustment for VAT, hip circumference and age (Table 3). Thus, independent of VAT, fasting TG levels were lower in females with NGT as compared to IGT or DM2.

**Table 2:** Pearson correlations coefficients (r) between fasting serum triglycerides and potential determinants.

	r	P-value
Age	0.08	0.022
Weight a	0.13	<0.001
Height	−0.02	0.644
BMI a	0.14	<0.001
Hip circumference	−0.09	0.005
VAT a	0.28	<0.001
SAT	0.03	0.478
Fasting plasma glucose^a^	0.15	<0.001
2 hour plasma glucose a	0.26	<0.001
Fasting plasma insulin a	0.21	<0.001
2 hour plasma insulin a	0.20	<0.001
Calculated insulin resistance (HOMA-IR)^a^	0.23	<0.001

a If appropriate, variables were transformed into their natural logarithm.

**Table 3:** Results from general linear models with fasting serum triglycerides as dependent variable.

Predictive variables in the model	B	SE	P
Glucose tolerance status:			<0.001
IGT vs. NGT	0.125	0.0346	<0.001
DM2 vs. NGT	0.079	0.0564	0.164
VAT	0.316	0.0377	<0.001
Hip circumference	−0.007	0.0015	<0.001
Age	−0.005	0.0014	<0.001

If appropriate, variables were transformed into their natural logarithm.

**Figure 1:**
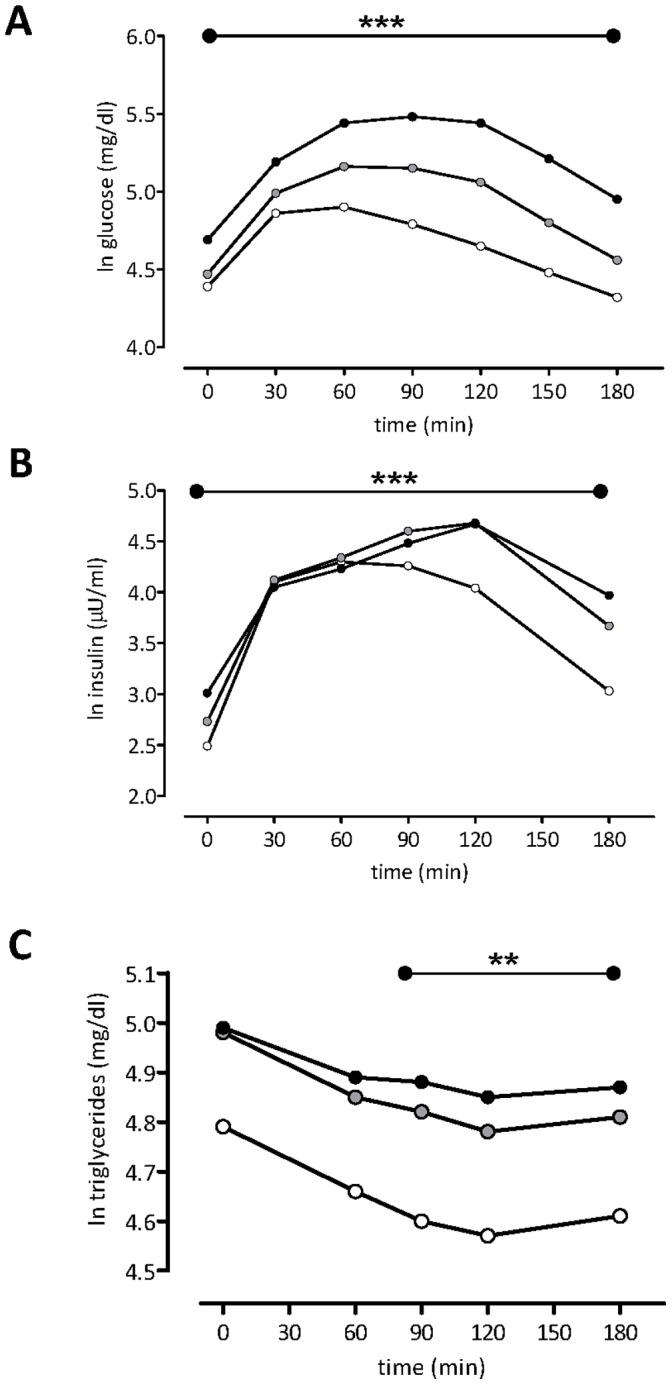
The post-load course of glucose-, insulin- and triglyceride alterations during 3 h after an oral glucose tolerance test (75 g) in obese females with normal glucose tolerance (NGT, open circles), pre-diabetes (IGT, gray circles) and newly diagnosed type 2 diabetes (DM2, black circles) . Glucose, see panel A; insulin, see panel B; triglycerides, see panel C. ***Statistical differences in the post-load course after an 75 g OGTT between NGT, IGT and DM2. **Statistical differences in the post-load course after an 75 g OGTT between IGT and DM2. See Information S1.

To explore determinants of non-fasting TG concentrations, the TG response after an 75 gram OGTT was analyzed using mixed modelling. Figure 1 illustrates the alterations in glucose, insulin and triglyceride concentrations during the course of the OGTT in the three groups. As expected, the glucose curve differs according to glucose tolerance status. Also, the insulin curve differs according to glucose tolerance status. Specifically, the initial post-load increase in insulin concentrations is faster and the duration more limited in females with NGT, as compared to DM2, whereas the insulin curve of females with IGT showed an intermediate behaviour. Finally, the post-load TG concentrations showed on average a decline in the various groups which seemed to be reduced in females with DM2. Females with IGT showed a similar post-load TG curve as females with NGT, although their initial TG concentrations, as well as the post-load TG concentrations were elevated as compared to females with NGT.

**Figure 2:**
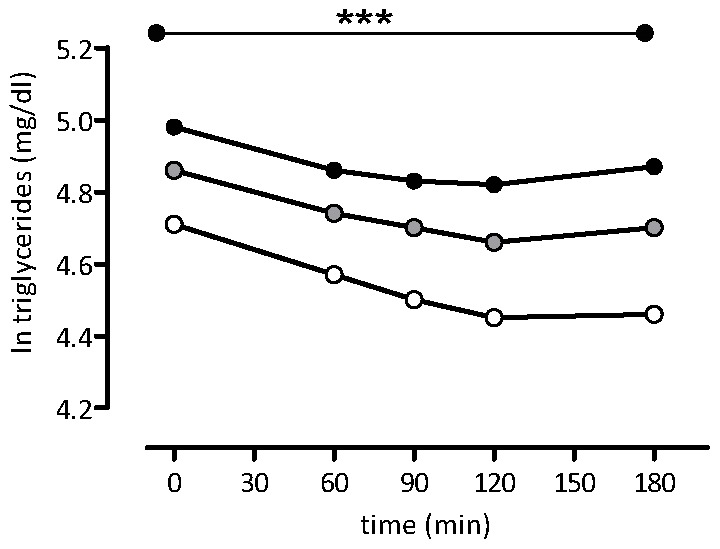
The post-load course of triglyceride alterations during 3 h after an 75 g OGTT in obese females are statistically different (***) according to tertiles of visceral adipose tissue (3rd tertile: females with largest amount of visceral adipose tissue). See supporting information S1.

Subsequently, additional determinants of TG concentrations after the OGTT were explored by mixed modelling analysis. This analysis identified VAT as the most important determinant of post-load TG concentrations. Accordingly, when distributing the participants into tertiles of VAT an almost equivalent distribution in post-load TG concentrations was observed among the three groups (Figure 2). Furthermore, the initial difference between IGT and DM2 lost statistical significance after adjustment for VAT.

Thus, fasting TG concentrations were lower in obese females with NGT as compared to IGT or NDM. This effect is independent of VAT. In contrast, the alterations in post-load TG response among obese females with NGT, IGT or DM2 were found to be dependent on the amount of VAT.

## Discussion

The present study displays an important link between fasting TG concentrations and glucose intolerance, as well as between the course of alterations in TG concentrations after an OGTT and the amount of VAT. These findings indicate that both glucose intolerance and increased amounts of VAT promote elevations in circulating TG concentrations, and thereby contribute to the development of lipotoxicity.

A limitation of the present study is that a general accepted systemic biomarker for lipotoxicity is not available. The use of NEFA has been heavily criticized: as adipose tissue mass expands, NEFA release per kilogram adipose tissue is downregulated, not increased [5]. NEFA concentrations might be modulated by altered function/expression of transporter proteins for fatty acids [19]. Based on former studies [**7**], [11], [12], we therefore used circulating TG levels as indicator for lipotoxicity. For non-fasting TG concentrations, the course of TG alterations after an OGTT was used instead of post-prandial TG levels after a standard mixed meal, as performing both tests was practically not feasible in more than 900 females. Another limitation is that results of the present study are not applicable to men or to females of African origin, as TG physiology is affected by sex steroids [14] and African descent [20]. Although a substantial number of females (n = 336) used oral contraceptive pills or hormonal replacement therapy, we assume that the sample size of this epidemiological study evens potential individual effects on glucose- or lipid metabolism, as users are randomly distributed. Finally, there was no power calculation with a priori established primary end points, and the data are obtained in a cross-sectional study, because a prospective evaluation of patients with glucose intolerance or DM2 *without* treatment cannot be regarded as ethical. Despite these limitations, the present study provides arguments – for the first time based on a large population at risk – that a reduced capacity to dispose serum TG is the most likely explanation for the well known association between “the hypertriglyceridemic waist (estimate of amount of visceral adipose tissue)” and “high risk of CHD” in glucose intolerance and DM2 [8]. I.e. lipotoxicity is confirmed to be a main issue for CHD [21], and is related to glucose intolerance and VAT.

Changes in visceral fat of Japanese men have been associated with incident metabolic syndrome, such that investigators concluded that, for prevention, a lifestyle should be adopted that does not increase visceral fat [22]. The same study showed also a positive association between changes in visceral fat and changes in fasting TG concentrations, confirming that visceral adipose tissue and TG are strongly related. The waist circumference has not only been associated with metabolic syndrome, DM2 and cardiovascular disease [8], but also with liver fibrosis in children [23]. The present findings confirm the notion of the importance of body shape for lipotoxicity and obesity-related metabolic complications [2], [7], and extent our knowledge by demonstrating that the amount of VAT is also a predominant regulator of *non-fasting* TG levels in obese females with or without a disturbed glucose metabolism. A dynamic study showed similar findings in detail in a limited number of obese males; they concluded that subjects with increased adiposity fails to appropriately upregulate meal fat-storage. This sign of maladaptation was thereafter suggested as the potential pathophysiologic basis of ectopic fat deposition in obesity [24].

Glucose intolerance in obese subjects has been associated with metabolically “inflexible” high basal intramuscular triglyceride concentrations [25], confirming the relationship between glucose intolerance and lipotoxicity. The deregulation of intramyocellular fatty acid metabolism appears to be one of the initial features of insulin resistance of the skeletal muscle, closely linked to a reduced capacity to dispose lipids properly [2], [26].

Collectively, the results of the present study provide a meaningful direction for future research into obesity-related metabolic complications. I.e. new studies should aim to reveal the relationship between, on the one hand, the capacity to dispose serum lipids, and, on the other hand, glucose intolerance and the amount of visceral adipose tissue. Important questions that remain to be addressed include the mechanism underlying the positive association between TG concentrations and the amount of VAT, as well as the understanding of the association between elevated fasting TG concentrations and visceral adipocyte size [6]? Finally, it remains to be established to what extent alterations in adipokine secretion from enlarged VAT contribute to the reduced capacity to dispose serum lipids [27].

In conclusion, fasting TG concentrations are associated with impaired glucose tolerance in obese Caucasian females, and the course of alterations in TG concentrations after an OGTT is determined by the amount of VAT. The findings suggest that glucose intolerance and the amount of VAT interfere with disposing of serum lipids.

## Supporting Information

Information S1 Results of mixed modelling analysis : glucose response according to glucose tolerance (Tables S1); Insulin response according to glucose tolerance (Tables S2); TG response according to glucose tolerance (Tables S3), and TG response according to tertiles of VAT (Tables S4).
(DOCX)
