# Supplementary material for: Glucose Intolerance and the Amount of Visceral Adipose Tissue Contribute to an Increase in Circulating Triglyceride Concentrations in Caucasian Obese Females
Source: PLoS One. 2012 Sep 28;7(9):e45145. doi: 10.1371/journal.pone.0045145 (PMC3460993; doi:10.1371/journal.pone.0045145)
Supplement: Information S1 — Results of mixed modelling analysis : glucose response according to glucose tolerance (Tables S1); Insulin response according to glucose tolerance (Tables S2); TG response according to glucose tolerance (Tables S3), and TG response according to tertiles of VAT (Tables S4). (DOCX) [file pone.0045145.s001.docx]

**Supporting information S1**

Tables S1 : Glucose (see Figure 1**,** panel A)

Tables S2 : Insulin (see Figure 1, panel B)

Tables S3 : Triglycerides (see Figure 1, panel C)

Tables S4 : Amount of VAT (see Figure 2)

**Figure 1, Panel A**

**Mixed model: Glucose response* according to glucose tolerance**

**TABLE S1.**Type III Tests of Fixed Effects

|  | P |
| --- | --- |
| Time | <0.001 |
| Glucose Tolerance Status | <0.001 |
| Interaction: Time - Glucose Tolerance Status | <0.001 |

**TABLE S1.** Estimate of fixed effects of glucose tolerance status on glucose response*.

|  | **Estimate** | **P** | **95% Confidence Interval** | | |
| --- | --- | --- | --- | --- | --- |
|  |  |  | **LowerBound** |  | **UpperBound** |
| **Estimate of glucose response* in DM2** |  |  |  |  |  |
| 0 min. (fasting glucose) | 4.69 | <0.001 | [4.64 | - | 4.74] |
| 30 min. | 0.50 | <0.001 | [0.44 | - | 0.56] |
| 60 min. | 0.74 | <0.001 | [0.68 | - | 0.80] |
| 90 min. | 0.79 | <0.001 | [0.73 | - | 0.84] |
| 120 min. | 0.75 | <0.001 | [0.69 | - | 0.81] |
| 150 min. | 0.51 | <0.001 | [0.45 | - | 0.57] |
| 180 min. | 0.26 | <0.001 | [0.20 | - | 0.32] |
| **Glucose response* in NGT compared to DM2** |  |  |  |  |  |
| 0 min. (fasting glucose) | -0.31 | <0.001 | [-0.36 | - | -0.26] |
| 30 min. | -0.03 | 0.356 | [-0.09 | - | 0.03] |
| 60 min. | -0.23 | <0.001 | [-0.30 | - | -0.17] |
| 90 min. | -0.38 | <0.001 | [-0.45 | - | -0.32] |
| 120 min. | -0.49 | <0.001 | [-0.55 | - | -0.42] |
| 150 min. | -0.42 | <0.001 | [-0.48 | - | -0.35] |
| 180 min. | -0.32 | <0.001 | [-0.39 | - | -0.26] |
| **Glucose response* in IGT compared to DM2** |  |  |  |  |  |
| 0 min. (fasting glucose) | -0.23 | <0.001 | [-0.29 | - | -0.17] |
| 30 min. | 0.03 | 0.398 | [-0.04 | - | 0.10] |
| 60 min. | -0.05 | 0.157 | [-0.12 | - | 0.02] |
| 90 min. | -0.10 | 0.004 | [-0.17 | - | -0.03] |
| 120 min. | -0.16 | <0.001 | [-0.22 | - | -0.09] |
| 150 min. | -0.18 | <0.001 | [-0.24 | - | -0.11] |
| 180 min. | -0.17 | <0.001 | [-0.23 | - | -0.10] |

*Estimate of glucose response* in females with DM2.
Comparison of glucose response* in females with NGT and females with IGT to females with DM2.*

**TABLE S1.** Estimate of fixed effects of glucose tolerance status on glucose response*.

|  | **Estimate** | **P** | **95% Confidence Interval** | | |
| --- | --- | --- | --- | --- | --- |
|  |  |  | **LowerBound** |  | **UpperBound** |
| **Estimate of glucose response* in IGT** |  |  |  |  |  |
| 0 min. (fasting glucose) | 4.46 | <0.001 | [4.44 | - | 4.49] |
| 30 min. | 0.53 | <0.001 | [0.50 | - | 0.56] |
| 60 min. | 0.70 | <0.001 | [0.66 | - | 0.73] |
| 90 min. | 0.68 | <0.001 | [0.65 | - | 0.72] |
| 120 min. | 0.59 | <0.001 | [0.56 | - | 0.63] |
| 150 min. | 0.34 | <0.001 | [0.30 | - | 0.37] |
| 180 min. | 0.09 | <0.001 | [0.06 | - | 0.13] |
| **Glucose response* in NGT compared to IGT** |  |  |  |  |  |
| 0 min. (fasting Gluc) | -0.08 | <0.001 | [-0.11 | - | -0.05] |
| 30 min. | -0.06 | 0.003 | [-0.10 | - | -0.02] |
| 60 min. | -0.19 | <0.001 | [-0.22 | - | -0.15] |
| 90 min. | -0.28 | <0.001 | [-0.32 | - | -0.24] |
| 120 min. | -0.33 | <0.001 | [-0.37 | - | -0.29] |
| 150 min. | -0.24 | <0.001 | [-0.28 | - | -0.20] |
| 180 min. | -0.16 | <0.001 | [-0.20 | - | -0.12] |

*Estimate of glucose response* in females with IGT.
Comparison of glucose response* in females with NGT to females with IGT.*

**TABLE S1.** Estimate of fixed effects of glucose tolerance status on glucose response*

|  | **Estimate** | **P** | **95% Confidence Interval** | | |
| --- | --- | --- | --- | --- | --- |
|  |  |  | **LowerBound** |  | **UpperBound** |
| **Estimate of glucose response* in NGT** |  |  |  |  |  |
| 0 min. (fasting glucose) | 4.38 | <0.001 | [4.37 | - | 4.40] |
| 30 min. | 0.47 | <0.001 | [0.45 | - | 0.49] |
| 60 min. | 0.51 | <0.001 | [0.49 | - | 0.53] |
| 90 min. | 0.40 | <0.001 | [0.38 | - | 0.42] |
| 120 min. | 0.26 | <0.001 | [0.24 | - | 0.29] |
| 150 min. | 0.10 | <0.001 | [0.08 | - | 0.12] |
| 180 min. | -0.06 | <0.001 | [-0.08 | - | -0.04] |

*Estimate of glucose response* in females with NGT.*

**TABLE S1.** Overview of estimated glucose response* in females with NGT, IGT and DM2.

| **Time (min.)** | **0** | **30** | **60** | **90** | **120** | **150** | **180** |
| --- | --- | --- | --- | --- | --- | --- | --- |
| **NGT** | 4.38 | 4.85 | 4.89 | 4.79 | 4.65 | 4.48 | 4.32 |
| **IGT** | 4.46 | 4.99 | 5.16 | 5.15 | 5.06 | 4.80 | 4.56 |
| **DM2** | 4.69 | 5.19 | 5.44 | 5.48 | 5.44 | 5.21 | 4.95 |

****After transformation of glucose levels into their natural logarithm.***

**Supporting Figure 1, Panel B**

**Mixed model: Insulin response* according to glucose tolerance**

**TABLE S2.** Type III Tests of Fixed Effects

|  | P |
| --- | --- |
| Time | <0.001 |
| Glucose Tolerance Status | <0.001 |
| Interaction: Time - Glucose Tolerance Status | <0.001 |

**TABLE S2.**Estimate of fixed effects of glucose tolerance status on insulin response*

|  | **Estimate** | **P** | **95% Confidence Interval** | | |
| --- | --- | --- | --- | --- | --- |
|  |  |  | **LowerBound** |  | **UpperBound** |
| **Estimate of insulin response* in DM2** |  |  |  |  |  |
| 0 min. (fasting insulins) | 3.01 | <0.001 | [2.81 | - | 3.21] |
| 30 min. | 1.03 | <0.001 | [0.83 | - | 1.24] |
| 60 min. | 1.22 | <0.001 | [1.02 | - | 1.42] |
| 90 min. | 1.46 | <0.001 | [1.25 | - | 1.67] |
| 120 min. | 1.66 | <0.001 | [1.46 | - | 1.86] |
| 180 min. | 0.95 | <0.001 | [0.75 | - | 1.15] |
| **Insulin response* in NGT compared to DM2** |  |  |  |  |  |
| 0 min. (fasting insulin) | -0.52 | <0.001 | [-0.73 | - | -0.31] |
| 30 min. | 0.57 | <0.001 | [0.36 | - | 0.78] |
| 60 min. | 0.59 | <0.001 | [0.38 | - | 0.80] |
| 90 min. | 0.31 | 0.006 | [0.09 | - | 0.53] |
| 120 min. | -0.11 | 0.318 | [-0.32 | - | 0.10] |
| 180 min. | -0.42 | <0.001 | [-0.63 | - | -0.20] |
| **Insulin response* in IGT compared to DM2** |  |  |  |  |  |
| 0 min. (fasting insulin) | -0.28 | 0.013 | [-0.50 | - | -0.06] |
| 30 min. | 0.36 | 0.002 | [0.13 | - | 0.58] |
| 60 min. | 0.39 | 0.001 | [0.16 | - | 0.62] |
| 90 min. | 0.40 | 0.001 | [0.16 | - | 0.64] |
| 120 min. | 0.29 | 0.013 | [0.06 | - | 0.52] |
| 180 min. | -0.02 | 0.883 | [-0.25 | - | 0.21] |

*Estimate of insulin response* in females with NDM.
Comparison of insulin response* in females with NGT and females with PreDM to females with NDM.*

**TABLE S2.** Estimate of fixed effects of glucose tolerance status on insulin response*.

|  | **Estimate** | **P** | **95% Confidence Interval** | | |
| --- | --- | --- | --- | --- | --- |
|  |  |  | **LowerBound** |  | **UpperBound** |
| **Estimate of insulin response* in IGT** |  |  |  |  |  |
| 0 min. (fasting insulin) | 2.73 | <0.001 | [2.63 | - | 2.84] |
| 30 min. | 1.39 | <0.001 | [1.28 | - | 1.50] |
| 60 min. | 1.61 | <0.001 | [1.50 | - | 1.72] |
| 90 min. | 1.86 | <0.001 | [1.75 | - | 1.98] |
| 120 min. | 1.95 | <0.001 | [1.84 | - | 2.06] |
| 180 min. | 0.93 | <0.001 | [0.82 | - | 1.04] |
| **Insulin response* in NGT compared to IGT** |  |  |  |  |  |
| 0 min. (fasting insulin) | -0.24 | <0.001 | [-0.37 | - | -0.11] |
| 30 min. | 0.21 | 0.001 | [0.09 | - | 0.34] |
| 60 min. | 0.20 | 0.002 | [0.07 | - | 0.33] |
| 90 min. | -0.09 | 0.177 | [-0.23 | - | 0.04] |
| 120 min. | -0.40 | <0.001 | [-0.53 | - | -0.27] |
| 180 min. | -0.40 | <0.001 | [-0.53 | - | -0.27] |

*Estimate of insulin response* in females with IGT.
Comparison of insulin response* in females with NGT to females with IGT.*

**TABLE S2.** Estimates of fixed effects of glucose tolerance status on insulin response*.

|  | **Estimate** | **P** | **95% Confidence Interval** | | |
| --- | --- | --- | --- | --- | --- |
|  |  |  | **LowerBound** |  | **UpperBound** |
| **Estimate of insulin response* in NGT** |  |  |  |  |  |
| 0 min. (fasting insulin) | 2.49 | <0.001 | [2.42 | - | 2.56] |
| 30 min. | 1.61 | <0.001 | [1.53 | - | 1.68] |
| 60 min. | 1.81 | <0.001 | [1.74 | - | 1.88] |
| 90 min. | 1.77 | <0.001 | [1.70 | - | 1.84] |
| 120 min. | 1.55 | <0.001 | [1.48 | - | 1.62] |
| 180 min. | 0.53 | <0.001 | [0.46 | - | 0.60] |

*Estimate of insulin response* in females with NGT.*

**TABLE S2.** Overview of estimated insulin response* in females with NGT, IGT and DM2.

| **Time (min.)** | **0** | **30** | **60** | **90** | **120** | **150** | **180** |
| --- | --- | --- | --- | --- | --- | --- | --- |
| **NGT** | 2.49 | 4.10 | 4.30 | 4.26 | 4.04 | - | 3.02 |
| **IGT** | 2.73 | 4.12 | 4.34 | 4.59 | 4.68 | - | 3.67 |
| **DM2** | 3.01 | 4.05 | 4.23 | 4.48 | 4.67 | - | 3.96 |

****After transformation of insulin levels into their natural logarithm.***

**Supporting Figure 1, Panel C**

**Mixed model: TG response* according to glucose tolerance**

**TABLE S3.** Type III Tests of Fixed Effects

|  | P |
| --- | --- |
| Time | <0.001 |
| Glucose Tolerance Status | <0.001 |
| Interaction: Time - Glucose Tolerance Status | <0.001 |

**TABLE S3.** Estimate of fixed effects of glucose tolerance status on triglyceride response*.

|  | **Estimate** | **P** | **95% Confidence Interval** | | |
| --- | --- | --- | --- | --- | --- |
|  |  |  | **Lower Bound** |  | **Upper Bound** |
| **Estimate of TG response* in DM2** |  |  |  |  |  |
| 0 min. (fasting TG) | 4.99 | <0.001 | [4.88 | - | 5.10] |
| 60 min. | -0.10 | <0.001 | [-0.13 | - | -0.07] |
| 90 min. | -0.12 | <0.001 | [-0.15 | - | -0.09] |
| 120 min. | -0.14 | <0.001 | [-0.17 | - | -0.11] |
| 180 min. | -0.12 | <0.001 | [-0.15 | - | -0.09] |
| **TG response* in NGT compared to DM2** |  |  |  |  |  |
| 0 min. (fasting TG) | -0.20 | <0.001 | [-0.32 | - | -0.09] |
| 60 min. | -0.03 | 0.098 | [-0.059 | - | 0.005] |
| 90 min. | -0.07 | <0.001 | [-0.099 | - | -0.034] |
| 120 min. | -0.07 | <0.001 | [-0.102 | - | -0.037] |
| 180 min. | -0.06 | <0.001 | [-0.092 | - | -0.027] |
| **TG response* in IGT compared to DM2** |  |  |  |  |  |
| 0 min. (fasting TG) | -0.01 | 0.834 | [-0.13 | - | 0.11] |
| 60 min. | -0.03 | 0.110 | [-0.063 | - | 0.006] |
| 90 min. | -0.05 | 0.009 | [-0.081 | - | -0.012] |
| 120 min. | -0.05 | 0.004 | [-0.085 | - | -0.016] |
| 180 min. | -0.05 | 0.004 | [-0.086 | - | -0.017] |

*Estimate of TG response* in females with DM2.
Comparison of TG response* in females with NGT and females with IGT to females with DM2.*

**TABLE S3.** Estimate of fixed effects of glucose tolerance status on triglyceride response*.

|  | **Estimate** | **P** | **95% Confidence Interval** | | |
| --- | --- | --- | --- | --- | --- |
|  |  |  | **Lower Bound** |  | **Upper Bound** |
| **Estimate of TG response* in IGT** |  |  |  |  |  |
| 0 min. (fasting TG) | 4.98 | <0.001 | [4.92 | - | 5.04] |
| 60 min. | -0.13 | <0.001 | [-0.14 | - | -0.11] |
| 90 min. | -0.16 | <0.001 | [-0.18 | - | -0.15] |
| 120 min. | -0.19 | <0.001 | [-0.21 | - | -0.18] |
| 180 min. | -0.17 | <0.001 | [-0.19 | - | -0.15] |
| **TG response* in NGT compared to IGT** |  |  |  |  |  |
| 0 min. (fasting TG) | -0.19 | <0.001 | [-0.26 | - | -0.12] |
| 60 min. | 0.001 | 0.918 | [-0.019 | - | 0.021] |
| 90 min. | -0.02 | 0.041 | [-0.040 | - | -0.001] |
| 120 min. | -0.02 | 0.052 | [-0.039 | - | 0.0002] |
| 180 min. | -0.01 | 0.407 | [-0.028 | - | 0.011] |

*Estimate of TG response* in females with IGT.*

*Comparison of TG response* in females with NGT to females with IGT.*

**TABLE S3.**Estimate of fixed effects of glucose tolerance status on TG response*.

|  | **Estimate** | **P** | **95% Confidence Interval** | | |
| --- | --- | --- | --- | --- | --- |
|  |  |  | **Lower Bound** |  | **Upper Bound** |
| **Estimate of TG response* in NGT** |  |  |  |  |  |
| 0 min. (fasting TG) | 4.79 | <0.001 | [4.75 | - | 4.82] |
| 60 min. | -0.13 | <0.001 | [-0.14 | - | -0.12] |
| 90 min. | -0.18 | <0.001 | [-0.19 | - | -0.17] |
| 120 min. | -0.21 | <0.001 | [-0.22 | - | -0.20] |
| 180 min. | -0.18 | <0.001 | [-0.19 | - | -0.17] |

*Estimate of TG response* in females with NGT.*

**TABLE S3.** Overview of estimated TG response* in females with NGT, IGT and DM2.

| **Time (min.)** | 0 | 30 | 60 | 90 | 120 | 150 | 180 |
| --- | --- | --- | --- | --- | --- | --- | --- |
| **NGT** | 4.79 | - | 4.66 | 4.60 | 4.57 | - | 4.61 |
| **IGT** | 4.98 | - | 4.85 | 4.82 | 4.78 | - | 4.81 |
| **DM2** | 4.99 | - | 4.89 | 4.88 | 4.85 | - | 4.87 |

****After transformation of TG levels into their natural logarithm.***

**Supporting Figure 2**

**Mixed model: TG response* according to the amount of VAT***

**TABLE S4.** Type III Tests of Fixed Effects

|  | P |
| --- | --- |
| Time | <0.001 |
| VAT* | <0.001 |
| Interaction: Time – VAT* | <0.001 |

**TABLE S4.** Estimate of fixed effects of VAT* on TG response*.

|  | **Estimate** | **P** | **95% Confidence Interval** | | |
| --- | --- | --- | --- | --- | --- |
|  |  |  | **Lower Bound** |  | **Upper Bound** |
| **Estimate of TG response* for median amount of VAT^+^** |  |  |  |  |  |
| 0 min. (fasting TG) | 4.85 | <0.001 | [4.82 | - | 4.88] |
| 60 min. | -0.13 | <0.001 | [-0.13 | - | -0.12] |
| 90 min. | -0.17 | <0.001 | [-0.18 | - | -0.16] |
| 120 min. | -0.20 | <0.001 | [-0.21 | - | -0.19] |
| 180 min. | -0.17 | <0.001 | [-0.18 | - | -0.16] |
| **Estimate of fixed effects of VAT*** |  |  |  |  |  |
| 0 min. (fasting TG) | 0.26 | <0.001 | [0.20 | - | 0.32] |
| 60 min. | 0.03 | 0.006 | [0.01 | - | 0.04] |
| 90 min. | 0.07 | <0.001 | [0.05 | - | 0.08] |
| 120 min. | 0.10 | <0.001 | [0.08 | - | 0.12] |
| 180 min. | 0.13 | <0.001 | [0.11 | - | 0.14] |

*^+^ Median amount of VAT: 133,6 cm^2^*

****After transformation of TG levels and VAT into their natural logarithm.***

**Mixed model: TG response* according to tertiles of VAT^+^**

**TABLE S4.** Type III Tests of Fixed Effects

|  | P |
| --- | --- |
| Time | <0.001 |
| VAT tertiles | <0.001 |
| Interaction: Time – VATtertiles | <0.001 |

**TABLE S4.** Estimate of fixed effects of the amount of VAT (tertiles) on TG response*.

|  | **Estimate** | **P** | **95% Confidence Interval** | | |
| --- | --- | --- | --- | --- | --- |
|  |  |  | **Lower Bound** |  | **Upper Bound** |
| **Estimate of TG response* in 3^th^tertile of VAT** |  |  |  |  |  |
| 0 min. (fasting TG) | 4.98 | <0.001 | [4.92 | - | 5.03] |
| 60 min. | -0.12 | <0.001 | [-0.13 | - | -0.10] |
| 90 min. | -0.14 | <0.001 | [-0.16 | - | -0.13] |
| 120 min. | -0.15 | <0.001 | [-0.17 | - | -0.14] |
| 180 min. | -0.11 | <0.001 | [-0.12 | - | -0.09] |
| **TG response* in 1^st^ compared to 3^th^tertile of VAT** |  |  |  |  |  |
| 0 min. (fasting TG) | -0.27 | <0.001 | [-0.34 | - | -0.19] |
| 60 min. | -0.03 | 0.015 | [-0.05 | - | -0.01] |
| 90 min. | -0.07 | <0.001 | [-0.09 | - | -0.05] |
| 120 min. | -0.11 | <0.001 | [-0.13 | - | -0.09] |
| 180 min. | -0.15 | <0.001 | [-0.17 | - | -0.12] |
| **TG response* in 2^nd^ compared to 3^th^tertile of VAT** |  |  |  |  |  |
| 0 min. (fasting TG) | -0.12 | 0.002 | [-0.19 | - | -0.05] |
| 60 min. | -0.004 | 0.730 | [-0.02 | - | 0.02] |
| 90 min. | -0.01 | 0.172 | [-0.04 | - | 0.01] |
| 120 min. | -0.04 | <0.001 | [-0.06 | - | -0.02] |
| 180 min. | -0.05 | <0.001 | [-0.07 | - | -0.03] |

*Estimate of TG response* in females with “highest” amount of VAT (3^th^tertile).
Comparison of TG response* in females with “lowest” (2^nd^tertile) and “intermediate” amount of VAT to females with “highest” amount of VAT.*

**TABLE S4.** Estimate of fixed effects of the amount of VAT (tertiles) on TG response*.

|  | **Estimate** | **P** | **95% Confidence Interval** | | |
| --- | --- | --- | --- | --- | --- |
|  |  |  | **Lower Bound** |  | **Upper Bound** |
| **Estimate of TG response* in 2^nd^tertile of VAT** |  |  |  |  |  |
| 0 min. (fasting TG) | 4.86 | <0.001 | [4.80 | - | 4.91] |
| 60 min. | -0.12 | <0.001 | [-0.13 | - | -0.10] |
| 90 min. | -0.16 | <0.001 | [-0.17 | - | -0.14] |
| 120 min. | -0.20 | <0.001 | [-0.21 | - | -0.18] |
| 180 min. | -0.16 | <0.001 | [-0.17 | - | -0.14] |
| **TG response* in 1^st^compared to 2^nd^tertile of VAT** |  |  |  |  |  |
| 0 min. (fasting TG) | -0.15 | <0.001 | [-0.22 | - | -0.07] |
| 60 min. | -0.02 | 0.036 | [-0.04 | - | 0.001] |
| 90 min. | -0.06 | <0.001 | [-0.08 | - | -0.03] |
| 120 min. | -0.06 | <0.001 | [-0.08 | - | -0.04] |
| 180 min. | -0.10 | <0.001 | [-0.12 | - | -0.07] |

*Estimate of TG response* in females with “intermediate” amount of VAT (2^nd^tertile).
Comparison of TG response* in females with “lowest” amount of VAT (1^st^tertile) to females with “intermediate” amount of VAT (2^nd^tertile).*

**TABLE S4.** Estimates of fixed effects of the amount of VAT (tertiles) on TG response*.

|  | **Estimate** | **P** | **95% Confidence Interval** | | |
| --- | --- | --- | --- | --- | --- |
|  |  |  | **Lower Bound** |  | **Upper Bound** |
| **Estimate of TG response* in 1^st^tertile of VAT** |  |  |  |  |  |
| 0 min. (fasting TG) | 4,71 | <0.001 | [4,66 | - | 4,76] |
| 60 min. | -0,14 | <0.001 | [-0,16 | - | -0,13] |
| 90 min. | -0,21 | <0.001 | [-0,23 | - | -0,20] |
| 120 min. | -0,26 | <0.001 | [-0,28 | - | -0,25] |
| 180 min. | -0,25 | <0.001 | [-0,27 | - | -0,24] |

*Estimate of TG response* in females with “lowest” amount of VAT (1^st^tertile).*

**TABLE S4.**Overview of estimated TG response* according to tertiles of VAT.

| **Time (min.)** | **0** | **30** | **60** | **90** | **120** | **150** | **180** |
| --- | --- | --- | --- | --- | --- | --- | --- |
| **1^st^tertile** | 4.71 | - | 4.57 | 4.50 | 4.45 | - | 4.46 |
| **2^nd^tertile** | 4.86 | - | 4.74 | 4.70 | 4.66 | - | 4.70 |
| **3^th^tertile** | 4.98 | - | 4.86 | 4.83 | 4.82 | - | 4.87 |

***^+^*Tertiles of VAT**

1^st^tertile: ≤ 109 cm^2^, median 86 cm^2^

2^nd^tertile: > 109 cm^2^ and ≤ 164 cm^2^, median 133.5 cm^2^

3^th^tertile:**>**164cm^2^, median 212 cm^2^

****After transformation of TG levels into their natural logarithm.***
